# Epidemiology of Wildlife Infectious Diseases

**DOI:** 10.3390/vetsci10050332

**Published:** 2023-05-05

**Authors:** Angela Magnet, Fernando Izquierdo

**Affiliations:** Facultad de Farmacia, Universidad San Pablo-CEU, CEU Universities, Urbanización Monteprincipe, 28660 Boadilla del Monte, Spain

The rise of infectious diseases in wildlife has become a severe concern recently, not only in relation to wildlife preservation but also for human health. Zoonotic diseases that jump from animals to humans have been emerging at an increasing rate, leading to outbreaks that have endangered global health. The concept of One Health recognizes the interconnection between the health of humans, animals, and the environment, emphasizing the need for collaboration across various disciplines to address emerging health challenges.

Infections can lead to the extinction or the decline of specific populations of vertebrates and invertebrates. The impact of infectious diseases on the wildlife population has been significant, leading to a loss of biodiversity and ecosystem services. Moreover, recently, the importance of human infectious diseases being transmitted to animals has strongly emphasized the importance of the One Health concept.

In this Special Issue, we focus on the epidemiology of different viruses, bacteria, and protozoa in different animal environments, including domestic, wild, aquatic, and zoological institutions. Understanding the epidemiology of these infectious diseases is the starting point for future risk management and conservation strategies. The link between wildlife infections and human health highlights the need to understand the epidemiology of infectious diseases in wildlife and develop strategies to prevent and control their spread.

A novel point of view on transmitting infectious diseases is presented by Koster et al. [1] in a review of how human pathogens affect great apes. This perspective is reinforced by Paungpin et al. [2], who discovered human influenza viruses in macaques in Thailand. The entrance of humans into wild environments for agriculture, forestry, and animal breeding leads to close contact with domestic and wild animals. Tucciarone et al. [3] studied the epidemiology of a typical poultry virus, Avian Metapneumovirus (aMPV), in wild birds, suggesting the latter’s susceptibility to this virus. The detection of aMPV in mallards emphasizes the importance of further studies that will deepen our understanding of the circulation of this virus to minimize the exchange of diseases of domestic animals with wild animals that can endanger their conservation and favor viral dissemination. This close contact between wild and domestic animals was shown in the study by Nurettin et al. [4], which presents similar seroprevalences of Crimean–Congo Hemorrhagic Fever in wild and domestic animals in Turkey.

Following this idea, understanding disease circulation in wild animals that are part of the human diet is critical to the prevention of zoonotic disease outbreaks. Additionally, monitoring the health of both wild and domestic animals is crucial to prevent the emergence and spread of infectious diseases. Fonti et al. [5] discuss how the hepatitis E virus circulates in wild deer, showing the zoonotic potential of consuming this game meat. At the same time, Baz-Gonzalez et al. [6] studied the infection of *Cryptosporidium* in wild rabbits hunted for human consumption, highlighting a potential risk to public health.

Most epidemiological studies focus on terrestrial animals, but we should not forget about aquatic creatures due to their specific characteristics. Felipe-Jiménez et al. [7] studied the epidemiology of Cetacean and Dolphin Morbillivirus in the Canary Islands, presenting new hosts for these viruses. This accentuated the importance of epidemiological studies that would help to prevent new epizootic events caused by these viruses. Finally, Marin et al. [8] studied the presence of resistant *Salmonella* in chelonians introduced in zoological institutions. This study presents the importance of establishing detection protocols for infectious agents for the entry of animals into these centers to prevent the spread of infectious diseases and protect the other resident animals or workers.

This Special Issue on the “Epidemiology of Wildlife Infectious Diseases” provides valuable insights into the epidemiology of wildlife infectious diseases and their potential implications for human and animal health. It also highlights the need for interdisciplinary collaborations that address emerging health challenges and develop effective strategies to prevent and control the spread of infectious diseases in wildlife.

Overall, the Special Issue underscores the urgent need for a One Health approach to address the complex challenges of emerging infectious diseases. It calls for collaborative efforts to promote the health and well-being of humans and animals and to safeguard the diversity of life on our planet.

## References

[1] Koster P.C., Lapuente J., Cruz I., Carmena D., Ponce-Gordo F. (2022). Human-Borne Pathogens: Are They Threatening Wild Great Ape Populations?. Vet. Sci..

[2] Paungpin W., Thongdee M., Ketchim N., Chaiwattanarungruengpaisan S., Saechin A., Sariya L., Kaewchot S., Puthavathana P., Wiriyarat W. (2022). Evidence of Influenza A Virus Infection in Cynomolgus Macaques, Thailand. Vet. Sci..

[3] Tucciarone C.M., Franzo G., Legnardi M., Pasotto D., Lupini C., Catelli E., Quaglia G., Graziosi G., Dal Molin E., Gobbo F. (2022). Molecular Survey on A, B, C and New Avian Metapneumovirus (aMPV) Subtypes in Wild Birds of Northern-Central Italy. Vet. Sci..

[4] Nurettin C., Engin B., Sukru T., Munir A., Zati V., Aykut O. (2022). The Seroprevalence of Crimean-Congo Hemorrhagic Fever in Wild and Domestic Animals: An Epidemiological Update for Domestic Animals and First Seroevidence in Wild Animals from Turkiye. Vet. Sci..

[5] Fonti N., Pacini M.I., Forzan M., Parisi F., Periccioli M., Mazzei M., Poli A. (2022). Molecular and Pathological Detection of Hepatitis E Virus in Roe Deer (*Capreolus capreolus*) and Fallow Deer (*Dama dama*) in Central Italy. Vet. Sci..

[6] Baz-Gonzalez E., Martin-Carrillo N., Garcia-Livia K., Foronda P. (2022). Molecular Detection of *Cryptosporidium cuniculus* in Rabbits (*Oryctolagus cuniculus*) from Tenerife, Canary Islands, Spain. Vet. Sci..

[7] Felipe-Jimenez I., Fernandez A., Arbelo M., Segura-Gothlin S., Colom-Rivero A., Suarez-Santana C.M., De La Fuente J., Sierra E. (2022). Molecular Diagnosis of Cetacean Morbillivirus in Beaked Whales Stranded in the Canary Islands (1999–2017). Vet. Sci..

[8] Marin C., Martin-Maldonado B., Cerda-Cuellar M., Sevilla-Navarro S., Lorenzo-Rebenaque L., Montoro-Dasi L., Manzanares A., Ayats T., Mencia-Gutierrez A., Jorda J. (2022). Antimicrobial Resistant Salmonella in Chelonians: Assessing Its Potential Risk in Zoological Institutions in Spain. Vet. Sci..

